# Economics, life history and international trade data for seven turtle species in Indonesian and Malaysian farms

**DOI:** 10.1016/j.dib.2020.106708

**Published:** 2020-12-31

**Authors:** Simon Kaae Andersen, Johanna Staerk, Elham Kalhor, Daniel J.D. Natusch, Rita da Silva, Beate Pfau, Dalia A. Conde

**Affiliations:** a Department of Biology, University of Southern Denmark, Campusvej 55, 5230 Odense M, Denmark; b Interdisciplinary Centre on Population Dynamics, University of Southern Denmark, 5230 Odense M, Denmark; c Species360, 7900 International Drive, Suite 1040 Bloomington, MN 55425 USA; d International Business and Entrepreneurship Research Group, Department of Marketing and Management, University of Southern Denmark, Campusvej 55, 5230 Odense M, Denmark; e Department of Biological Sciences, Macquarie University, North Ryde, NSW 2109 Australia; f EPIC Biodiversity, Frogs Hollow, NSW 2550, Australia; g German Society for Herpetology and Herpetoculture (DGHT), Vogelsang 27, 31020 Salzhemmendorf, Germany

**Keywords:** Breeding facilities, Captive breeding, CITES, Confiscation, Farm economics, Testudines, Wildlife trade

## Abstract

We collected data on the trade of seven turtle and tortoise species endemic to Indonesia and Malaysia (*Amyda cartilaginea, Batagur borneoensis, Cuora amboinensis, Carettochelys insculpta, Heosemys annandalii, Heosemys grandis*, and *Heosemys spinosa*). The data on those species included: operations costs of three breeding farms and one export facility; species life-history traits; and species international legal trade and confiscation data. We collected data for the facilities (one in Malaysia and three in Indonesia) using site visits and a semi-structured questionnaire. We conducted a literature review to compile relevant information on species’ life-history traits to estimate breeding viability. We downloaded species-specific data on international trade from the Convention on International Trade in Endangered Species of Wild Fauna and Flora (CITES) Trade Database for the exporting countries (Malaysia and Indonesia) for 2000–2015. We compared legal trade with confiscation data obtained from CITES. The data in this article can provide insights into the operations of turtle breeding farms in Southeast Asia. These data can be used as a reference for the inspection of breeding farms and for legislative bodies to determine whether captive breeding for select turtle species is feasible.

## Specifications Table

**Table:** 

Subject	Environmental Science: Management, Monitoring, Policy and Law
Specific subject area	Management and international trade of turtles in Indonesia and Malaysia
Type of data	Table
How data were acquired	Field visits and standardized questionnaires, literature review, data download from online databases processed using R version 1.2.5033 [1]
Data format	Raw
	Analyzed
	Filtered
Parameters for data collection	We selected seven turtle species that are reported to be captive-bred or wild-caught in Southeast Asia, are in international trade, and are globally threatened with extinction. Data were collected anonymously to protect facility operators and to reduce possible bias in survey answers.
Description of data collection	Data on the facilities were obtained by the inspection of four breeding farms (one registered facility in Malaysia, and three facilities in Indonesia) and interviews with facility operators using a standardized questionnaire. We conducted a literature search for species’ life-history traits and downloaded data on international commercial trade from the Convention on International Trade in Endangered Species of Wild Fauna and Flora (CITES): UNEP-WCMC CITES Trade Database for the exporting countries (Malaysia and Indonesia) from 2000 to 2015. We compared data from legal trade with confiscation data obtained from CITES, CoP17 Doc annex 1 [2].
Data source location	City/Town/Region: Jakarta, West Jakarta, Bogor
	Countries: Malaysia and Indonesia
	Other data: Online databases and scientific literature
Data accessibility	Within this article, on Dryad and also on the Species360 Open Data Portal [3] Direct URL to data in Dryad: https://datadryad.org/stash/share/FoaXdRwNx1p4uowKsrLBmZcv0hR8fDvQAL93NgPn_no, Direct URL to data in the Species360 Open Data Portal: https://conservation.species360.org/open-data/

## Value of the Data

• Turtles are traded as pets and also for consumption (including for meat, eggs, and traditional medicine). The data in this article are important for understanding the degree to which turtle breeding farms can supply demand for otherwise wild-caught turtles and whether wildlife laundering may be occurring.
• The data can benefit wildlife trade authorities, conservation organizations, researchers investigating wildlife breeding farms, and legislative bodies such as the Convention on International Trade in Endangered Species of Wild Fauna and Flora (CITES). The data are relevant for those investigating trade routes of exotic wildlife in Southeast Asia.
• The data can be used to estimate the profitability of turtle breeding and likely prices for the end consumer. Thus, the data can inform whether claims of captive breeding are legitimate and economically sustainable.
• The data also provide standardized information on life-history traits that can be used in further studies of turtle biology, evolution, and conservation.

## 1 Data Description

Table 1 provides an overview of the seven species studied (*Amyda cartilaginea, Batagur borneoensis, Cuora amboinensis, Carettochelys insculpta, Heosemys annandalii, Heosemys grandis*, and *Heosemys spinosa*), including their International Union for Conservation of Nature (IUCN) Red List status, CITES Appendix listing, listing in the Annexes of the European Union Wildlife Trade Regulations, and countries of occurrence. Table 2 provides an overview of the four inspected facilities and the species found there. Table 3 shows the sale price (USD) for turtles in the pet, meat, and breeding stock markets, as reported by the facilities in Indonesia and Malaysia. The supplemental data file 1 contains detailed information on the operations and economics of the inspected facilities. The supplemental data file 2 contains the full list of questions asked of the facility owners.

**Table 1:** Species IUCN Red List status, CITES Appendix, EU Wildlife Trade Regulations Annex, and countries of occurrence. IUCN categories are listed as Vulnerable (VU), Endangered (EN), and Critically Endangered (CR). All species were assessed by IUCN in 2000 except for *Carettochelys insculpta*, which was assessed in 2017.

Species	IUCN category	CITES Appendix	EU Annex	Distribution
*Amyda cartilaginea*	VU	ll	B	Brunei Darussalam, Cambodia, India, Indonesia, Lao People's Democratic Republic, Malaysia, Myanmar, Singapore, Thailand, Vietnam
*Batagur borneoensis*	CR	ll	B	Brunei Darussalam, Indonesia, Malaysia, Thailand
*Carettochelys insculpta*	EN	ll	B	Australia, Indonesia, Papua New Guinea
*Cuora amboinensis*	VU	ll	B	Bangladesh, Bhutan, Brunei Darussalam, Cambodia, India, Indonesia, Lao People's Democratic Republic, Malaysia, Myanmar, Philippines, Singapore, Thailand, Timor-Leste, Vietnam
*Heosemys annandalii*	EN	ll	B	Cambodia, Lao People's Democratic Republic, Malaysia, Myanmar, Thailand, Vietnam
*Heosemys grandis*	VU	ll	B	Cambodia, Lao People's Democratic Republic, Malaysia, Myanmar, Singapore, Thailand, Vietnam
*Heosemys spinosa*	EN	ll	B	Brunei Darussalam, Indonesia, Malaysia, Myanmar, Philippines, Singapore, Thailand

**Table 2:** Overview of the inspected facilities, including species present at each facility. The facility in Central Jakarta reported that they did not breed turtles but legally exported wild individuals under the quota system.

	Indonesia			
	Central Jakarta	Bogor	West Jakarta	Malaysia
Species	Export of wild turtles	Captive breeding	Captive breeding	Captive breeding

*Amyda cartilaginea*	X	X	X	X
*Batagur borneoensis*		X		X
*Carettochelys insculpta*		X	X	
*Cuora amboinensis*	X	X	X	X
*Heosemys annandalii*				X
*Heosemys grandis*				X
*Heosemys spinosa*		X	X

**Table 3:** Selling prices for turtles in the pet, meat, and breeding stock markets as reported by facilities in Indonesia and Malaysia. Prices shown in US Dollars (USD), converted where necessary from EUR, SGD, and RMB according to May 2020 exchange rates. Prices are shown for adult individuals, hatchlings, and kg of turtle, where relevant. NA: Not Applicable, the species is not being sold for this purpose.

	Pets			Meat		Breeding stock	
Indonesia	Japan (individuals)	USA (individuals)	EU (individuals)	Singapore (kg)	China (kg)	NA	
Amyda cartilaginea	$35–45	$25–45	$25	$6–7	$11		NA
Batagur borneoensis	$100–150	$100–150	NA	NA	NA		NA
Carettochelys insculpta	$100–300	$100–500	NA	NA	NA		NA
Cuora amboinensis	$10–15	$10–16	NA	NA	$11		NA
Heosemys spinosa	$40–50	$40–125	NA	NA	NA		NA

Malaysia	Japan (hatchlings)	China (per kg of large individuals)	China (hatchlings)	Singapore (kg)	China (kg)		China (individuals)

Amyda cartilaginea	NA	NA	$28–35	$15–21	$28–30		NA
Batagur borneoensis	$300	NA	NA	NA	NA		NA
Cuora amboinensis	$26	NA	$28–35	NA	$22–25		$42–49
Heosemys annandalii	NA	NA	$56	NA	NA		NA
Heosemys grandis	NA	$56–63	42	NA	$28–35		NA

The supplemental file 3 contains detailed data on the CITES-regulated international trade of the seven species as well as data on confiscations (2000–2015) extracted from the UNEP-WCMC CITES Trade Database. Tables 4 and 5 summarize legal trade and confiscation data, respectively.

**Table 4:** Numbers of live individuals traded in the international commercial trade (2000–2015) under different CITES source codes [4] from the UNEP-WCMC CITES Trade Database.

	Trade sources				
**Species**	Wild	Captive bred	Farmed	Ranched	Total
Amyda cartilaginea	359,127	650	1100	0	360,877
Batagur borneoensis	15,650	1736	553	1000	18,939
Carettochelys insculpta	0	30	57	0	87
Cuora amboinensis	877,284	26,502	5738	1000	910,524
Heosemys annandalii	10,628	5581	0	25,010	41,219
Heosemys grandis	41,144	7741	3	44,512	93,400
Heosemys spinosa	33,043	971	247	0	34,261

**Table 5:** Summary of confiscations form the CITES CoP17 Doc Annex 1 document [2], for the seven study species; Including the number of live individuals confiscated and the corresponding number of confiscation events. For parts and derivatives, only the number of confiscation events is shown.

	Live animals		Parts and derivatives
Species	# specimens	# events	# events
*Amyda cartilaginea*	7704	14	16
*Batagur borneoensis*	81	2	0
*Carettochelys insculpta*	29,692	26	2
*Cuora amboinensis*	20,772	37	8
*Heosemys annandalii*	353	22	2
*Heosemys grandis*	1292	29	7
*Heosemys spinosa*	709	7	0

The supplemental file 4 contains detailed data on the life-history traits of the seven turtle species. Table 6 provides an overview of the completeness of life-history data for each species.

**Table 6:** Number of life-history traits available for the seven study species out of 13 collated variables. Numbers in brackets show total possible number of variables per category.

Species	Reproduction (7)	Survival (5)	Weight (1)	Total (13)
*Amyda cartilaginea*	7	4	1	12
*Batagur borneoensis*	6	3	1	10
*Carettochelys insculpta*	3	1	1	5
*Cuora amboinensis*	7	4	1	12
*Heosemys annandalii*	7	5	1	13
*Heosemys grandis*	7	5	1	13
*Heosemys spinosa*	7	4	1	12

## 2 Experimental Design, Materials and Methods

### 2.1 Field data

We obtained data on farm operations, economics, and production from four Southeast Asian turtle breeding and export facilities for seven species endemic to the region (*Amyda cartilaginea, Batagur borneoensis, Cuora amboinensis, Carettochelys insculpta, Heosemys annandalii, Heosemys grandis*, and *Heosemys spinosa*). We selected species based on the following criteria: i) if they were listed in one of the CITES Appendices ii) if they were assessed as globally threatened according to the IUCN Red List (i.e. vulnerable, endangered or critically endangered) and iii) if commercial captive breeding has been questioned either in the literature or by experts. However, we only obtained data for those species that were present at the facilities visited at the time of the study; thus, this was a significant factor. We selected the facilities non-randomly based on the availability and the willingness of the owners to be interviewed. One facility was located in Malaysia, which is the only one of its kind representing the industry of commercial breeding of turtles in the country. The other three facilities were located in Indonesia, two in Jakarta (Central and West Jakarta), while the third is located in Bogor. The facility in Central Jakarta reported that they did not breed turtles but only exported wild animals. In Indonesia, we do not know if the visited facilities are representative of the country's industry.

From June to October 2019, we conducted face-to-face interviews with facility owners to gather economic data on traded species. Facility owners often kept Multiple species within a single facility, but usually under separate management regimes. Despite all facilities keeping and breeding turtles, the three Indonesian facilities also exported wild-caught specimens of the same species. Facilities varied in size, depending on location and the number of species produced. Some facilities bred a variety of taxa (e.g., snakes, lizards, and turtles) and only dedicated ∼600 m^2^ to turtle production. Two of the facilities in Indonesia were much larger, with 10,000 m^2^ dedicated to turtle production. Enclosure sizes also varied among facilities. The smallest Indonesian facility kept a small number of turtles (e.g., up to six specimens in enclosures that averaged 16 m^2^). The largest facility in Malaysia kept hundreds of turtles in ponds measuring 325 m^2^.

To interview facility owners, we used a targeted sampling strategy and a semi-structured approach with a standardized series of questions. Questions were designed to give an overview of the key economic parameters required to determine the economic feasibility of turtle production based on farm inputs and outputs. The data included export prices, export costs (including transport boxes and shipping costs), running costs for facilities (such as utilities and staff), and the cost of permits required to breed, keep, and export turtles in Malaysia and Indonesia. These data were augmented with questions about species-specific biological parameters (e.g., time to maturity, growth rates, survival, and reproduction). Not all facility owners were able to answer all questions.

For Indonesia, we further obtained data on export quotas (i.e., the number of wild-sourced individuals that can be legally sold) [5] and data on current SATS-LN (CITES permit; Surat Angkut Tumbuhan dan Satwa Luar Negeri) and SATS-DN (Indonesian Domestic Transport Permit or Interprovincial Transport Permit; Surat Angkut Tumbuhan dan Satwa Dalam Negeri) permit requirements and costs [6].

### 2.2 CITES legal trade data and confiscations

We downloaded information on the legal international commercial trade of Testudines from 2000 to 2015 from the UNEP-WCMC CITES Trade Database on the 10th of September 2019 [7]. Although this dataset is arguably incomplete (see a review on this topic by Robinson and Sinovas [8]), it is the most complete dataset openly available for trade analyses of the kind. We included data for the following species: *A. cartilaginea, B. borneoensis, C. amboinensis, C. insculpta, H. annandalii, H. grandis*, and *H. spinosa*. We excluded re-exports (i.e. records where the country of origin is different from the country of export) to avoid double-counting shipments. When both exported and imported quantities were stated but differed, we used the larger of the two to avoid underestimating trade levels. We included trade from the following CITES source codes, W (wild), C (bred in captivity), F (born in captivity), and R (ranched), defined in the CITES source code booklet [4].

We extracted data on confiscations from illegal trade from the CITES CoP17 Doc Annex 1 document [2]. We extracted seizure content from all major confiscation events (defined as a confiscation of >3000 individuals or 3000 kg of products of tortoises and freshwater turtles) that included at least one individual from any of the seven studied species.

### 2.3 Life-history data

We performed a literature search to compile life-history traits relevant to breeding viability for *A. cartilaginea, B. borneoensis, C. amboinensis, C. insculpta, H. annandalii, H. grandis*, and *H. spinosa*. The data include a total of 233 records spread across seven reproductive variables, five survival variables, and one mass variable. Reproductive variables include age of first reproduction, percentage of females reproducing each year, number of clutches per female, number of eggs per clutch, mating season, egg-laying season, and incubation time. Survival data include maximum lifespan, sex ratio, hatching success, mortality in first year, and mortality in years beyond the first year. Mass data include the body mass of adult turtles. We obtained the majority of the data (169 records) from the TRAFFIC (2013) inspection manual for use in commercial reptile breeding [9]. This source, however, did not include *B. borneoensi*s and *C. insculpta*. We included data from Guntoro (2011 [10], 2012 [11], 2013 [12]) for *B. borneoensis* (16 records) and data from Doody et al. (2003) [13] for *C. insculpta* (10 records). We supplemented the dataset with information from the Species360 database on turtle and tortoise demographic traits [3] (38 records).

## Ethics Statement

We have obtained informed consent from all the participants. We informed all participants that they could leave at any time and/or refuse to answer any question, and there was no coercion or pressure to answer questions. Our research involved negligible risk for human participants, since no personal data was inquired or collected, all questions were strictly related to business, and were collected completely anonymously.

## CRediT Author Statement

DAC developed the conception and design of the study; SKA, JS, RS, DJDN, and DAC participated in the acquisition of data and all authors participated in the curation and analysis of data. SKA, JS, EK and DAC drafted the first version of the manuscript and all authors contributed to the following versions. The final version of the manuscript to be published was approved by all authors.

## Declaration of Competing Interest

The authors declare that they have no known competing financial interests or personal relationships which have, or could be perceived to have, influenced the work reported in this article.

## Appendix Supplementary materials

Image, application 1

Image, application 2

Image, application 3

Image, application 4

## Footnotes

Supplementary material associated with this article can be found in the online version at doi:10.1016/j.dib.2020.106708.
